# Correction: Politicization of COVID-19 health-protective behaviors in the United States: Longitudinal and cross-national evidence

**DOI:** 10.1371/journal.pone.0263100

**Published:** 2022-01-21

**Authors:** Wolfgang Stroebe, Michelle R. vanDellen, Georgios Abakoumkin, Edward P. Lemay, William M Schiavone, Maximilian Agostini, Jocelyn J. Bélanger, Ben Gützkow, Jannis Kreienkamp, Anne Margit Reitsema, Jamilah Hanum Abdul Khaiyom, Vjolica Ahmedi, Handan Akkas, Carlos A. Almenara, Mohsin Atta, Sabahat Cigdem Bagci, Sima Basel, Edona Berisha Kida, Allan B. I. Bernardo, Nicholas R. Buttrick, Phatthanakit Chobthamkit, Hoon-Seok Choi, Mioara Cristea, Sára Csaba, Kaja Damnjanović, Ivan Danyliuk, Arobindu Dash, Daniela Di Santo, Karen M Douglas, Violeta Enea, Daiane Gracieli Faller, Gavan Fitzsimons, Alexandra Gheorghiu, Ángel Gómez, Ali Hamaidia, Qing Han, Mai Helmy, Joevarian Hudiyana, Bertus F. Jeronimus, Ding-Yu Jiang, Veljko Jovanović, Željka Kamenov, Anna Kende, Shian-Ling Keng, Tra Thi Thanh Kieu, Yasin Koc, Kamila Kovyazina, Inna Kozytska, Joshua Krause, Arie W. Kruglanksi, Anton Kurapov, Maja Kutlaca, Nóra Anna Lantos, Cokorda Bagus Jaya Lemsmana, Winnifred R. Louis, Adrian Lueders, Najma Iqbal Malik, Anton Martinez, Kira O. McCabe, Jasmina Mehulić, Mirra Noor Milla, Idris Mohammed, Erica Molinario, Manuel Moyano, Hayat Muhammad, Silvana Mula, Hamdi Muluk, Solomiia Myroniuk, Reza Najafi, Claudia F. Nisa, Boglárka Nyú, Paul A. O’Keefe, Jose Javier Olivas Osuna, Evgeny N. Osin, Joonha Park, Gennaro Pica, Antonio Pierro, Jonas Rees, Elena Resta, Marika Rullo, Michelle K. Ryan, Adil Samekin, Pekka Santtila, Edyta Sasin, Birga M. Schumpe, Heyla A. Selim, Michael Vicente Stanton, Samiah Sultana, Robbie M. Sutton, Eleftheria Tseliou, Akira Utsugi, Jolien Anne van Breen, Caspar J. Van Lissa, Kees Van Veen, Alexandra Vázquez, Robin Wollast, Victoria Wai-Lan Yeung, Somayeh Zand, Iris Lav Žeželj, Bang Zheng, Andreas Zick, Claudia Zúñiga, N. Pontus Leander

There is an error in affiliation 54 for author Adil Samekin. The correct affiliation 54 is: School of Liberal Arts, M. Narikbayev KAZGUU University, Nur-Sultan, Kazakhstan.
