# Supplementary material for: Politicization of COVID-19 health-protective behaviors in the United States: Longitudinal and cross-national evidence
Source: PLoS One. 2021 Oct 20;16(10):e0256740. doi: 10.1371/journal.pone.0256740 (PMC8528320; doi:10.1371/journal.pone.0256740)
Supplement: S1 Table. Unstandardized coefficients for the paths from baseline political orientation to perceived health risk (a) and from perceived risk to health-protective behaviors (b) at five time points, Study 1 — (DOCX) [file pone.0256740.s001.docx]

| Time point | Political Orientation to Perceived Risk | | | Perceived Risk to WHO Virus Mitigation Behaviors | | | |  |
| --- | --- | --- | --- | --- | --- | --- | --- | --- |
|  | *a* | *SE* | CI | | *b* | *SE* | CI | |
| March 10 | .065 | .015 | .034, .094 | | .064 | .030 | .006, .122 | |
| March 20 | .070 | .018 | .035, .105 | | .043 | .033 | -.022, .107 | |
| March 28 | .085 | .017 | .052, .119 | | .110 | .035 | .042, .178 | |
| April 11 | .050 | .018 | .014, .086 | | .118 | .039 | .042, .193 | |
| June 16 | .062 | .018 | .026, .098 | | .137 | .052 | .038, .240 | |

*Note*. CI = 95% bootstrap confidence interval
