# Supplementary material for: Politicization of COVID-19 health-protective behaviors in the United States: Longitudinal and cross-national evidence
Source: PLoS One. 2021 Oct 20;16(10):e0256740. doi: 10.1371/journal.pone.0256740 (PMC8528320; doi:10.1371/journal.pone.0256740)
Supplement: S2 Table — (DOCX) [file pone.0256740.s002.docx]

|  | Baseline *n* | Follow-up *n* |
| --- | --- | --- |
| **Canada** | **1531** | **266-443** |
| China | 1572 | 10-34 |
| France | 1753 | 311-499 |
| Germany | 1685 | 371-530 |
| **Greece** | **2870** | **534-980** |
| **Indonesia** | **2407** | **139-372** |
| Italy | 1996 | 393-673 |
| **Netherlands** | **2992** | **649-940** |
| Philippines | 1527 | 103-242 |
| **Republic of Serbia** | **2118** | **381-761** |
| **Romania** | **2696** | **260-516** |
| Russia | 1437 | 162-345 |
| Saudi Arabia | 1460 | 84-189 |
| South Africa | 1421 | 205-382 |
| South Korea | 1447 | 19-54 |
| **Spain** | **3156** | **807-1333** |
| Turkey | 1822 | 135-380 |
| Ukraine | 1427 | 214-260 |
| United Kingdom | 1902 | 457-655 |
| U.S. | 10923 | 1441-4166 |

*Note*. Sample sizes varied for each outcome (perceived risk, perceived efficacy of social distancing and mask wearing, WHO virus mitigation behaviors, wearing a face covering, and intentions to be vaccinated). Averaging across waves for each individual creates the largest possible sample size for each analysis. Countries in bold were selected for specific comparisons with the U.S.
