# Supplementary material for: Politicization of COVID-19 health-protective behaviors in the United States: Longitudinal and cross-national evidence
Source: PLoS One. 2021 Oct 20;16(10):e0256740. doi: 10.1371/journal.pone.0256740 (PMC8528320; doi:10.1371/journal.pone.0256740)
Supplement: S3 Table — (DOCX) [file pone.0256740.s003.docx]

|  | *M* (*SD)* | Wearing a Face Covering | Intentions to be Vaccinated | |
| --- | --- | --- | --- | --- |
| Virus Mitigation Behaviors | 1.75 (1.19) | .32 | .28 |  |
| Wearing a Face Covering | 3.76 (1.47) |  | .16 |  |
| Intentions to be Vaccinated | 0.91 (1.18) |  |  |  |
