# Supplementary material for: Politicization of COVID-19 health-protective behaviors in the United States: Longitudinal and cross-national evidence
Source: PLoS One. 2021 Oct 20;16(10):e0256740. doi: 10.1371/journal.pone.0256740 (PMC8528320; doi:10.1371/journal.pone.0256740)
Supplement: S4 Table — (DOCX) [file pone.0256740.s004.docx]

| Outcome | Baseline Day | Age | Gender | Education | Political Orientation | Location | Political Orientation * Location Interaction |
| --- | --- | --- | --- | --- | --- | --- | --- |
| **Baseline** |  |  |  |  |  |  |  |
| Perceived Risk | 762.54*** | 3.45 | 101.64*** | 394.32*** | 92.32*** | 0.06 | 45.58*** |
| Perceived Severity of Infection | 0.28 | 1062.00*** | 653.27*** | 50.56*** | 69.23*** | .014 | 67.52*** |
| WHO Virus Mitigation Behaviors | 392.41*** | 100.91*** | 1296.85*** | 123.69*** | 105.29*** | 0.31 | 67.15*** |
| **Follow-up** |  |  |  |  |  |  |  |
| Perceived Risk | 92.28*** | 79.06*** | 39.96*** | 64.62*** | 103.04*** | 0.01 | 63.96*** |
| Perceived Efficacy of Mask Wearing | 7.08*** | 48.69*** | 13.61*** | 1.34 | 52.38*** | 0.03 | 49.10*** |
| Perceived Efficacy of Social Distancing | 15.09*** | 61.33*** | 22.97*** | 11.78*** | 123.29*** | 0.04 | 76.19*** |
| WHO Virus Mitigation Behaviors | 8.29** | 6.48* | 34.63*** | 0.58 | 79.37*** | 0.05 | 61.89*** |
| Wearing a Face Covering |  | 41.75*** | 59.19*** | 13.61*** | 107.69*** | 0.15 | 87.61*** |
| Vaccine Intentions |  | 0.02 | 37.28*** | 85.31*** | 140.30*** | 0.15 | 88.51*** |

**p*<.05, ***p*<.01, ****p*<.001
