# Supplementary material for: Politicization of COVID-19 health-protective behaviors in the United States: Longitudinal and cross-national evidence
Source: PLoS One. 2021 Oct 20;16(10):e0256740. doi: 10.1371/journal.pone.0256740 (PMC8528320; doi:10.1371/journal.pone.0256740)
Supplement: S5 Table — (DOCX) [file pone.0256740.s005.docx]

| Date | U.S. *r(*N) | Non-U.S. *r(*N) | U.S. vs. Non-U.S. Comparison |
| --- | --- | --- | --- |
| **Perceived Risk** |  |  |  |
| Wave 1 | -.25*** (541) | .01 (983) | *F* = 18.24*** ƞ^2^= .011 |
| Wave 2 | -.18*** (2679) | -.07*** (3527) | *F* = 12.46*** ƞ^2^= .002 |
| Wave 3 | -.17*** (1863) | -.06*** (3633) | *F* = 9.67** ƞ^2^= .002 |
| Wave 4 | -.22*** (1361) | -.08*** (6608) | *F* = 17.18*** ƞ^2^= .002 |
| Wave 5 | -.15*** (1037) | -.08*** (6252) | *F =* 3.18 ƞ^2^ < .001 |
| Wave 7 | -.20*** (603) | -.06*** (4672) | *F* = 8.88** ƞ^2^= .002 |
| Wave 9 | -.17*** (746) | -.05*** (4066) | *F* = 7.52** ƞ^2^= .002 |
| Wave 11 | -.22*** (772) | -.05** (4145) | *F* = 18.23*** ƞ^2^= .004 |
| Wave 12 | -.19*** (693) | -.06*** (3617) | *F* = 7.80** ƞ^2^= .002 |
| **Efficacy of Social Distancing** | | | |
| Wave 1 | -.19*** (2679) | -.03 (3528) | *F* = 27.20*** ƞ^2^= .004 |
| Wave 2 | -.23*** (1864) | -.04* (3636) | *F* = 33.27*** ƞ^2^= .004 |
| Wave 3 | -.25*** (1362) | -.04** (6608) | *F* = 50.20*** ƞ^2^= .006 |
| **Efficacy of Wearing a Mask/Face Covering** | | | |
| Wave 6 | -.13*** (966) | .08*** (5592) | *F* = 38.23*** ƞ^2^= .006 |
| Wave 8 | -.19*** (838) | .06*** (4516) | *F* = 44.06*** ƞ^2^= .008 |
| Wave 10 | -.11* (552) | .07*** (3598) | *F* = 16.10*** ƞ^2^= .004 |
| Wave 12 | -.17*** (693) | .03 (3618) | *F* = 22.35*** ƞ^2^= .005 |
| **WHO Virus Mitigation Behaviors** | | | |
| Wave 4 | -.19*** (1362) | -.02** (6610) | *F* = 34.81*** ƞ^2^= .004 |
| Wave 11 | -.28*** (772) | .02 (4144) | *F* = 53.11*** ƞ^2^= .011 |
| Wave 12 | -.26*** (693) | .03 (3621) | *F* = 42.98*** ƞ^2^= .010 |
| **Vaccine Intentions** | | | |
| Wave 4 | -.31*** (1362) | -.06*** (6534) | *F* = 65.37*** ƞ^2^= .008 |
| Wave 11 | -.29*** (772) | -.05** (4115) | *F* = 33.71*** ƞ^2^= .007 |
| Wave 12 | -.30*** (693) | -.09*** (3597) | *F* = 20.14*** ƞ^2^= .005 |
| **Wearing a Mask** | | | |
| Wave 6 | -.23*** (888) | .06*** (5050) | *F* = 46.34*** ƞ^2^= .008 |
| Wave 8 | -.32*** (807) | .02 (4314) | *F* = 49.03*** ƞ^2^= .009 |
| Wave 10 | -.21*** (530) | .00 (3417) | *F* = 11.37*** ƞ^2^= .003 |
| Wave 12 | -.30*** (693) | -.02* (3329) | *F* = 11.84*** ƞ^2^= .003 |

**p* <.05, ***p*<.01, ****p*<.001
