# Supplementary material for: Politicization of COVID-19 health-protective behaviors in the United States: Longitudinal and cross-national evidence
Source: PLoS One. 2021 Oct 20;16(10):e0256740. doi: 10.1371/journal.pone.0256740 (PMC8528320; doi:10.1371/journal.pone.0256740)
Supplement: S6 Table — (DOCX) [file pone.0256740.s006.docx]

|  | IV to Mediator  *b* (CI) | | Mediator to DV  *b* (CI) | Direct Effect  *b* (CI) | | Indirect Effect  *b* (CI) | Index of Moderated Mediation |
| --- | --- | --- | --- | --- | --- | --- | --- |
| **Political Orientation – Perceived Risk—WHO Virus Mitigation Behaviors** | | | | | | | |
| Wave 4 |  |  |  | |  | | |
| US | -.248 (-.306, -.190) | | .064 (.019, .109) | -.016 (-.031, -.116) | | -.016 (-.031, -.002) | .010 (.005, .017) |
| Non-US | -.105 (-.136, -.075) | | .069 (.050, .088) | -.010 (-.034, .015) | | -.007 (-.011, -.004) |  |
| Wave 11 |  |  |  | |  | | |
| US | -.257 (-.337, -.177) | | .144 (.076, .212) | -.278 (-.356, -.200) | | -.037 (-.063, -.015) | .032 (.017, .048) |
| Non-US | -.056 (-.095, -.016) | | .147 (.116, .178) | .037 (-.003, .078) | | -.008 (-.015, -.002) |  |
| Wave 12 |  |  |  | |  | | |
| US | -.197 (-.277, -.117) | | .100 (.025, .175) | -.270 (-.352, -.189) | | -.020 (-.042, - .002) | .017 (.005, .031) |
| Non-US | -.069 (-.110, -.209) | | .124 (.088, .159) | .044 (-.000, .088) | | -.009 (-.015, -.003) |  |
| **Political Orientation – Perceived Risk—Willingness to be Vaccinated** | | | | | | | |
| Wave 4 |  | |  |  | |  |  |
| US | -.260 (-.311, -.209) | | .180 (.134, .225) | -.260 (-.311, -.209) | | -.044 (-.062, -.029) | .021 (.010, .032) |
| Non-US | -.111 (-.142, -.080) | | .140 (.119, .161) | -.051 (-.078, -.024) | | -.016 (-.021, -.011) |  |
| Wave 11 |  | |  |  | |  |  |
| US | -.257 (-.227, -.117) | | .219 (.157, .281) | -.251 (-.322, -.179) | | -.056 (-.083, -.033) | .032 (.017, .049) |
| Non-US | -.058 (-.098, -.019) | | .143 (.115, .171) | -.052 (-.089, -.016) | | -.008 (-.015, -.002) |  |
| Wave 12 |  | |  |  | |  |  |
| US | -.197 (-.277, -.117) | | .115 (.049, .181) | -.278 (-.350, -.206) | | -.023 (-.042, -.001) | .015 (004, .027) |
| Non-US | -.069 (-.110, -.029) | | .108 (.077, .139) | -.094 (-.133, -.056) | | -.007 (-.013, -.003) |  |
