# Supplementary material for: Politicization of COVID-19 health-protective behaviors in the United States: Longitudinal and cross-national evidence
Source: PLoS One. 2021 Oct 20;16(10):e0256740. doi: 10.1371/journal.pone.0256740 (PMC8528320; doi:10.1371/journal.pone.0256740)
Supplement: S7 Table — (DOCX) [file pone.0256740.s007.docx]

|  | IV to Mediator  *b* (CI) | | Mediator to DV  *b* (CI) | Direct Effect  *b* (CI) | Indirect Effect  *b* (CI) | Index of Moderated Mediation |
| --- | --- | --- | --- | --- | --- | --- |
| **Political Orientation – Perceived Risk—Wearing a Mask** | | | | | | |
| Wave 6 |  |  |  | |  | |
| US | -.201 (-.297, -.104) | | .153 (.078, .228) | -.274 (-.363, -.185) | -.031 (-.055, -.012) | .009 (-.001, .020) |
| Non-US | -.105 (-.148, -.061) | | .073 (.033, .114) | .078 (.025, .131) | -.008 (-.014, -.003) |  |
| Wave 8 |  |  |  | |  | |
| US | -.281 (-.393, -.170) | | .228 (.141, .315) | -.285 (-.383, -.186) | -.064 (-.108, -.028) | .034 (.011, .058) |
| Non-US | -.087 (-.135, -.040) | | .159 (.117, .201) | .013 (-.042, .068) | -.014 (-.023, -.006) |  |
| Wave 10 |  |  |  | |  | |
| US | -.183 (-.306, - .061) | | .200 (.107, .293) | -.161 (-.269, -.053) | -.037 (0.074, -.011) | .024 (<.001,.050) |
| Non-US | -.051 (-.102, .001) | | .176 (.130, .222) | .025 (-.035, .086) | -.009 (-.019, .001) |  |
| Wave 12 |  |  |  | |  | |
| US | -.206 (-.288, -.125) | | .097 (.047, .147) | -.192 (-.246, -.138) | -.020 (-.038, -.006) | .024 (.007, .043) |
| Non-US | -.072 (-.114, -.029) | | .180 (.141, .219) | -.016 (-.064, - .005) | -.013 (-.021, -.005) |  |
| **Political Orientation – Perceived Efficacy— Wearing a Mask** | | | | | | |
| Wave 6 |  |  |  | |  | |
| US | -.121 (-.192, -.049) | | .328 (.266, .391) | -.214 (-.282, -.146) | -.040 (-.069, -.015) | .121 (.074, .166) |
| Non-US | .107 (.072, .142) | | .549 (.518, .581) | .031 (-.009, .071) | .059 (.039, .079) |  |
| Wave 8 |  | |  |  |  |  |
| US | -.226 (-.302, -.150) | | .350 (.291, .408) | -.255 (-.319, -.190) | -.079 (-.114, -.047) | .171 (.121, .220) |
| Non-US | .087 (.048, .125) | | .565 (.533, .597) | -.023 (-.063, .018) | .049 (.027, .070) |  |
| Wave 10 |  | |  |  |  |  |
| US | -.103 (-.197, -.009) | | .373 (.301, .444) | -.173 (-.251, -.094) | -.039 (-.081, .001) | .109 (.049, .170) |
| Non-US | .100 (.057, .143) | | .548 (.511, .584) | -.049 (-.096, -.003) | .055 (.031, .078) |  |
| Wave 12 |  | |  |  |  |  |
| US | -.184 (-.261, -.106) | | .280 (.232, .329) | -.160 (-.210, -.111) | -.051 (-.084, -.024) | .117 (.067, .168) |
| Non-US | .042 (.001, .084) | | .530 (.494, .566) | -.053 (-.097, -.009) | .022 (-.001, .046) |  |

*Note*. Perceived risk was assessed in the prior wave (with the exception of Wave 12) whereas beliefs that masks protect oneself was assessed concurrently.
