# Supplementary material for: Politicization of COVID-19 health-protective behaviors in the United States: Longitudinal and cross-national evidence
Source: PLoS One. 2021 Oct 20;16(10):e0256740. doi: 10.1371/journal.pone.0256740 (PMC8528320; doi:10.1371/journal.pone.0256740)
Supplement: S8 Table — (DOCX) [file pone.0256740.s008.docx]

| **Variable** | **U.S.** | **Spain** | **Romania** | **Serbia** | **Netherlands** | | **Indonesia** | **Greece** | **Canada** |
| --- | --- | --- | --- | --- | --- | --- | --- | --- | --- |
|  | *r*  (N) | *r*  (N) | *r*  (N) | *r*  (N) | *r*  (N) | *r*  (N) | | *r*  (N) | *r*  (N) |
| **Baseline** |  |  |  |  |  |  | |  |  |
| Perceived Risk  (Total r) | **-.13**  **(10784)** | -.04  (3150) | .05  (2655) | **-.10**  **(2110)** | **-.08**  **(2985)** | -.04  (2395) | | **-.10**  **(2856)** | **-.10**  **(1529)** |
| Perceived Risk  (Partial r) | **-.11**  **(10784)** | -.02  (3125) | .00  (2655) | **-.06**  **(2096)** | -.04  (2857) | -.03  (2344) | | **-.07**  **(2842)** | -.04  (1501) |
| Perceived Severity  (Total r) | **-.08 (10784)** | .04  (3152) | .02  (2655) | .00  (2109) | .02  (2857) | .02  (2344) | | .01  (2842) | .03  (1501) |
| Perceived Severity  (Partial r) | **-.08**  **(10784)** | .03  (3125) | .02  (2655) | .01  (2096) | -.01  (2857) | .02  (2344) | | .02  (2842) | .00  (1501) |
| WHO Virus Mitigation Behaviors  (Total r) | **-.14**  **(10784)** | -.06  (3153) | .02  (2655) | **-.07**  **(2111)** | **-.11**  **(2857)** | .02  (2344) | | -.01  (2842) | **-.07**  **(1501)** |
| WHO Virus Mitigation Behaviors  (Partial r) | **-.11**  **(10784)** | -.04  (3125) | .02  (2655) | **-.04**  **(2096)** | **-.06**  **(2857)** | .03  (2344) | | .03  (2842) | **-.04**  **(1501)** |
| **Follow-Up** |  |  |  |  |  |  | |  |  |
| Perceived Risk  (Total r) | **-.19**  **(4166)** | -.01  (1333) | .00  (516) | **-.10**  **(761)** | -.06  (940) | -.04  (372) | | -.07  (980) | **-.09**  **(443)** |
| Perceived Risk  (Partial r) | **-.16**  **(892)** | 04  (611) | .15  (124) | -.01  (276) | -.02  (390) | .21  (52) | | -.12  (270) | -.04  (169) |
| Efficacy of Wearing a Mask (Total r) | **-.17**  **(1489)** | .02  (820) | .04  (292) | **-.11**  **(393)** | .06  (676) | **.01**  **(155)** | | .06  (562) | **-.16**  **(279)** |
| Efficacy of Wearing a Mask (Partial r) | **-.17**  **(892)** | .03  (611) | .08  (124) | **-.19**  **(276)** | .09  (390) | **-.16**  **(52)** | | .07  (270) | **-.17**  **(169)** |
| Efficacy of Social Distancing (Total r) | **-.22 (3576)** | -.08  (1087) | .06  (306) | **-.16**  **(625)** | -.09  (649) | -.03  (264) | | .21  (647) | **-.15 (341)** |
| Efficacy of Social Distancing  (Partial r) | **-.21 (892)** | -.06  (611) | -.08  (124) | **-.11**  **(276)** | -.09  (390) | -.08  (52) | | .22  (270) | **-.23 (169)** |
| Virus Mitigation Behaviors (Total r) | **-.23 (1811)** | -.02  (902) | .09  (347) | -.05  (550) | -.05  (699) | -.01  (202) | | .07  (570) | -.07 (324) |
| Virus Mitigation Behaviors (Partial r) | **-.22**  **(892)** | -.03  (611) | -.01  (124) | -.14  (276) | -.13  (390) | -.01  (52) | | .05  (270) | -.13 (169) |
| Wearing a Mask (Total r) | **-.28**  **(1441)** | .07  (807) | .06  (260) | -.04  (381) | -.01  (699) | -.03  (146) | | .11  (534) | **-.23 (266)** |
| Wearing a Mask  (Partial r) | **-.24**  **(892)** | .02  (611) | -.03  (124) | -.07  (276) | -.07 (390) | .06  (52) | | .11  (270) | **-.17 (169)** |
| Vaccine Intentions  (Total r) | **-.32**  **(1811)** | -.03  (902) | .02  (260) | **-.20**  **(547)** | -.10  (699) | .13  (139) | | .04  (562) | **-.29 (324)** |
| Vaccine Intentions (Partial r) | **-.27**  **(892)** | .00  (611) | .03  (124) | **-.19**  **(276)** | -.07  (390) | .14  (52) | | .02  (270) | **-.31 (169)** |

Notes. Within row, we compared the correlations between political orientation and each outcome in the U.S. to each other country. Country correlations in bold did not differ from the U.S. correlations (*p* <.05). Partial correlations reflect the association between political orientation and each outcome controlling for demographic factors (i.e., age, education, gender, and date of baseline survey completion).
