# Supplementary material for: Politicization of COVID-19 health-protective behaviors in the United States: Longitudinal and cross-national evidence
Source: PLoS One. 2021 Oct 20;16(10):e0256740. doi: 10.1371/journal.pone.0256740 (PMC8528320; doi:10.1371/journal.pone.0256740)
Supplement: Attachment — Submitted filename: Response to Reviewers.docx [file pone.0256740.s011.docx]

Reviewer 1

***“I would like to first acknowledge the authors efforts to address my many points and their attempt to simplify the results.

Their revised analyses are now much more streamlined, but they have sacrificed some nuance in the process. I am relatively sceptical of their approach of averaging responses across waves. I’m willing to accept the proposed stability of political orientation over time. But there are inherent assumptions in averaging outcomes and mediators across waves. Fortunately the authors mitigate this to some extent by retaining the original analyses in the supplementary material, so the motivated reader can examine the effects by wave more closely and see they are relatively consistent over time. This is not the approach I would have taken, but the authors are clear about the choices they have made, and I believe their conclusions can be drawn from the results they report, so I’m willing to (begrudgingly) accept this approach.”***

We appreciate the reviewer’s candor. We carefully considered many possibilities for presenting our results, especially given their longitudinal consistency. The choice we ultimately made would only have been possible given this consistency and, as the reviewer notes, we were able to use supplemental materials to report the full details of each analysis to allow readers access to this information.

***“I believe the manuscript to be publishable but note the following minor points for further revision.

I made a previous recommendation that the authors outline how future research might circumvent some of the limitations of the current study. Although their response states that they have added this to the discussion I was not able to spot it (apologies if I have missed it).”***

Although we had added to the discussion section statements that addressed our limitations, we did not explicitly state what we would have done in future studies. We have now added these explicit statements.  ***“I think there may be an error in the column labels of Table S4. It currently appears that the waves were alternatingly conducted in US and non-US samples, with the table values being separated by location by row as well. I would guess that the US/Non-Us labelling in the columns is an error?”***

Thank you for catching this mistake. That row was relevant to the Table we adapted to create Table S4. We have removed it and hope the information is now more helpful.  ***“Other than that point of confusion, I found this table to be very informative for understanding the structure of dataset. I would recommend that this be included in the main text – especially as the average column represents the final variables used in the main text analyses. It would help the reader to understand what is going on a bit better. If the authors are hesitant about the size of the table then perhaps a very simple table or diagram outlining which variables were included in which waves could suffice?”***

We are glad this table was helpful and we moved it to the main text as the reviewer suggested.  ***“I would also clarify in the main text that averages were calculated for any participant that provided a response for a given variable at least one relevant wave. That is, a given participants average score on, say, perceived efficacy of face coverings could be the average of between 1 and 4 responses, depending on how many of the relevant waves they participated in. At least that is how I interpret the revised approach – if I’m wrong then then the nature of how averages were calculated should still be clarified.”***

The reviewer’s interpretation of how averages were calculated is correct; we have added text to the document to increase this clarity.  ***“A refence on page 10 (Franz and Dhanani (2021)) is incorrectly formatted.”***

Because this reference was not an endote (i.e., we referred to the study as the subject of the sentence), reference formatting was not relevant. We have modified the sentence to make the reference consistent with others in the text.  ***“Table 4 – clarify the nature of the comparison column, I assume this is the F result and effect size for the PO*location interaction term in the GLM. But it is not clear in the table or text.”***

Table 4 is now included as Table 5. We have added text to clarify what the F and eta squared reports represent.  ***“In Table 5 I assume the indirect effect CI is based on bootstrapping, please note in the table note or text the number of samples. I would also recommend reiterating that the PO variable was standardised in the table note.”***

Table 5 is now Table 6. The bootstrapping approach and number of samples is already reported (see Page 29). We have added a reminder that the political orientation variable was standardized.

***“Reviewer #2: The revisions have strengthened the manuscript and addressed my major concerns and those of the other reviewer. I leave it to the editors to render a decision about the availability of the data and any lingering concerns this may pose with respect to journal policy. In any case, the revised manuscript is theoretically and technically sound and makes a valuable contribution to the burgeoning literature on this topic. I support publication in its current form.”***

We thank this reviewer for their time in reviewing the manuscript.
